# Supplementary material for: Surface Microbiome Profiling of Dental Elevators Using Third‐Generation Sequencing: Implications for Infection Control in Dental Practice
Source: Microbiologyopen. 2025 Dec 8;14(6):e70178. doi: 10.1002/mbo3.70178 (PMC12685768; doi:10.1002/mbo3.70178)
Supplement: Supplementary file 1 — customer_backup. [file MBO3-14-e70178-s001.zip › customer_backup/customer_backup/taxa_summary/krona/groups/treat/M05.Krona.html]

Javascript must be enabled to view this page.

members
magnitude
magnitudeUnassigned

M05.krona

57394

57394

2

0

0

0

0

0

0

0

0

0

0

0

0

0

0

0

2

2

2

2

0

0

0

0

0

0

0

1

1

0

0

0

0

0

0

0

0

0

0

0

0

0

0

33

15

0

0

0

0

4

4

2

2

1

1

0

0

0

1

1

0

0

11

3

3

3

1

1

1

0

0

0

0

0

0

0

1

1

1

3

2

2

0

0

1

1

0

0

0

3

3

3

0

0

18

0

0

0

0

0

0

0

18

1

1

1

3

3

3

2

2

2

6

6

6

6

6

6

978

54

54

0

0

0

54

29

29

0

20

8

12

0

0

5

5

21

0

0

0

0

0

0

0

0

0

0

0

0

0

0

0

0

0

0

0

0

0

0

0

0

0

0

0

0

0

0

0

0

0

0

0

0

0

21

21

21

21

0

0

0

0

0

0

0

0

903

903

60

0

0

0

0

0

0

0

0

0

0

1

1

59

59

0

0

0

0

0

0

0

0

0

843

0

0

0

0

0

843

843

0

0

0

0

0

0

0

0

0

0

0

0

0

0

0

0

0

0

0

0

0

0

0

0

0

0

0

0

0

0

0

0

0

0

0

0

0

0

0

2

2

2

2

2

2

0

0

0

0

0

0

0

0

1

1

1

0

0

0

0

0

0

1

1

1

0

0

0

0

0

0

30

2

2

2

0

0

1

1

0

0

0

1

0

1

1

1

1

1

1

27

27

27

1

1

0

26

0

0

21

0

5

0

0

0

0

0

39

7

7

0

0

0

7

7

7

0

0

0

0

0

0

0

0

0

0

0

0

0

0

0

0

32

15

12

10

1

8

1

2

0

2

0

0

3

3

3

0

0

0

0

0

0

0

0

0

0

16

0

0

0

0

0

0

0

0

0

0

0

0

0

0

0

0

0

0

0

0

0

0

0

0

16

16

4

12

1

1

0

0

0

0

1

1

0

0

0

0

0

0

0

0

0

0

14447

925

43

43

43

43

15

1

1

1

11

1

1

0

1

1

0

0

0

9

0

9

0

3

3

3

18

18

12

0

12

6

6

0

0

12

0

0

0

12

1

1

0

0

1

1

10

10

0

0

0

4

4

0

0

0

0

4

0

0

0

4

0

0

0

0

0

0

0

92

92

1

1

5

1

0

2

1

1

0

0

2

2

0

0

0

0

0

0

0

0

0

0

2

0

1

0

1

0

0

0

1

1

20

0

17

1

2

0

0

0

57

0

0

0

0

0

0

0

0

0

0

0

0

3

1

0

0

1

21

11

0

15

0

0

1

4

0

0

0

0

0

4

2

0

0

2

0

0

0

0

0

0

0

0

0

519

12

0

0

4

0

4

0

0

0

0

0

1

1

0

7

2

0

5

0

0

0

41

0

0

0

5

3

0

0

1

1

0

0

0

36

36

0

0

0

0

0

0

1

1

1

3

3

3

0

0

0

284

0

0

0

0

0

0

0

78

74

4

1

1

0

84

84

0

0

0

0

0

1

1

24

2

10

10

2

0

0

0

0

0

96

0

0

0

0

1

0

1

0

0

91

0

3

0

0

8

2

2

0

0

4

4

2

2

0

0

0

0

0

2

2

2

0

0

0

0

0

0

7

1

1

0

0

0

0

0

0

0

6

1

4

0

1

5

3

3

0

2

2

0

55

2

0

2

2

2

25

25

2

1

1

0

0

0

0

0

0

0

0

24

1

23

4

4

0

4

97

9

9

0

0

0

0

2

0

0

2

0

0

24

2

4

0

0

6

0

2

0

0

0

10

0

0

0

0

0

6

0

6

0

0

0

0

0

0

1

1

55

55

2

2

0

0

2

2

58

1

0

0

1

1

57

2

1

1

0

0

11

0

11

1

1

0

0

0

2

2

41

41

0

0

0

0

0

0

1

1

0

0

0

0

1

1

0

0

0

0

0

0

0

0

0

0

0

0

0

0

0

0

1

1

1

1

0

0

0

0

0

0

0

0

0

0

0

0

16

16

16

16

0

0

0

56

56

0

0

1

0

1

53

53

1

1

0

0

0

0

0

0

0

0

0

1

0

1

0

0

0

0

0

0

0

0

0

0

0

0

0

0

0

6

1

1

1

0

0

0

0

0

0

0

0

0

4

4

4

0

0

0

0

0

0

0

0

0

1

1

1

82

82

0

0

1

1

0

0

0

0

0

0

0

0

0

0

0

0

0

0

0

1

1

0

0

0

0

1

1

0

0

0

0

0

43

41

0

2

0

0

16

0

5

11

18

18

0

0

2

0

2

0

13522

0

0

0

0

0

0

0

0

0

0

0

0

3

3

3

3

0

0

0

10800

21

0

0

21

21

0

0

0

0

0

0

0

51

0

0

0

0

0

0

0

51

51

0

0

4

4

4

1

1

1

8

6

6

2

2

2538

1

1

2

2

2397

2397

126

1

112

8

5

12

0

0

0

0

0

7

0

1

0

3

0

0

1

0

0

0

6

0

0

1

1

4

1

3

0

0

0

0

0

1

0

1

0

0

0

122

0

0

0

0

0

0

0

0

0

0

122

0

0

122

0

0

4861

0

0

0

0

0

0

0

4861

0

4861

0

6

6

6

3178

58

58

0

0

0

0

1

1

10

10

1

1

4

1

2

1

11

0

11

0

0

84

0

84

0

0

1

0

1

427

1

426

0

0

0

22

0

22

2559

2559

0

0

4

3

3

1

0

1

7

5

1

1

0

3

3

1

1

2

2

0

2

0

0

0

0

0

0

0

0

0

0

0

0

0

0

0

0

0

0

0

0

1

1

1

1

0

0

0

0

0

0

0

0

0

0

0

0

0

0

0

442

0

0

0

275

0

0

0

0

0

0

0

119

119

156

0

0

18

138

0

0

0

0

0

0

0

0

0

0

0

0

0

0

0

0

0

0

18

18

18

110

110

0

0

99

11

39

0

0

0

0

0

0

0

29

29

0

0

10

10

0

0

0

0

0

0

0

0

0

0

0

0

0

1

1

0

0

0

0

1

1

0

0

0

0

0

0

0

67

0

0

0

67

0

0

0

0

67

0

67

0

0

0

0

2152

2

1

1

1

1

0

0

0

2

0

0

0

2

2

0

0

0

402

275

275

42

42

14

14

71

71

0

0

0

0

272

272

272

0

0

0

0

0

0

1474

0

0

0

0

1

1

34

34

1058

0

74

0

984

381

342

39

0

0

0

0

0

0

0

0

0

0

0

0

0

0

0

0

0

0

0

0

0

0

0

0

0

0

0

0

0

0

0

0

0

45

8

2

2

1

0

1

4

4

1

1

0

0

0

37

8

8

0

0

0

0

0

0

0

4

1

0

2

1

25

1

1

3

0

0

0

10

1

1

1

0

2

5

1

0

0

0

1

1

1

0

0

0

0

0

0

0

0

0

0

0

0

0

0

0

2

2

2

0

2

0

0

0

0

0

0

0

0

0

0

0

0

0

0

0

0

0

0

0

0

0

0

0

1

0

0

0

0

0

0

1

1

1

0

0

0

0

0

0

0

0

0

0

0

0

18

6

6

0

0

0

6

0

0

0

0

0

0

1

1

1

0

1

1

1

0

0

3

2

1

0

12

0

0

0

0

0

0

0

0

0

0

0

0

0

0

0

0

0

7

5

0

0

0

0

0

0

1

0

1

4

2

0

2

0

0

0

0

0

0

0

1

0

0

0

1

0

0

1

0

1

0

0

1

1

0

0

0

0

5

5

5

0

0

2

3

0

0

0

0

0

0

0

0

0

0

0

0

0

0

0

0

0

0

0

0

0

3

3

3

3

3

0

1

0

2

0

0

0

0

0

0

3165

0

0

0

0

0

3164

76

0

0

0

76

0

0

0

3

0

1

2

1

1

0

0

0

5

0

5

0

1

1

0

0

56

0

0

0

1

0

7

0

48

0

2

0

0

1

0

1

0

0

0

0

1

0

0

1

2

2

0

0

2

0

2

0

3

3

0

0

0

0

0

0

0

0

101

0

0

0

72

0

0

3

3

0

0

0

0

61

0

40

21

0

0

0

0

0

0

2

0

2

0

0

0

0

0

0

0

0

0

0

0

0

0

0

0

0

1

1

0

0

0

0

5

5

14

14

14

0

0

0

0

0

0

15

0

0

0

0

15

15

0

0

2915

0

0

0

0

0

0

0

0

0

0

0

195

0

0

1

1

48

48

146

146

203

0

0

0

0

0

137

137

0

0

0

0

0

0

66

0

13

12

3

1

3

0

5

29

0

31

31

31

36

0

0

0

0

36

0

7

29

7

0

0

7

7

0

0

0

177

177

0

4

1

1

135

2

0

0

0

0

1

1

13

2

0

1

16

797

13

13

733

716

17

51

1

23

6

5

0

16

588

0

0

0

1

0

1

7

0

1

6

0

0

2

2

0

0

0

0

319

0

47

0

0

79

0

0

0

0

0

0

171

0

0

0

0

22

259

0

0

0

0

19

13

133

0

0

13

0

0

0

81

0

0

0

0

0

0

0

0

589

589

0

57

81

451

0

0

0

0

0

292

292

292

1

0

0

0

1

1

1

0

0

0

0

0

0

0

0

0

0

0

0

0

0

0

0

0

0

0

71

3

0

0

0

0

0

0

3

0

3

0

57

0

0

1

0

1

0

56

56

0

0

1

0

0

0

0

1

0

1

0

0

0

0

0

0

0

0

3

2

2

1

1

0

0

0

0

7

0

0

1

0

0

0

0

0

1

0

0

0

0

0

0

0

0

6

1

0

0

0

1

1

0

0

0

2

1

0

0

0

0

0

0

0

0

0

0

0

0

0

0

1

1

0

0

0

1

1

1

0

0

0

0

0

0

0

0

0

0

0

0

0

0

0

9

8

0

0

0

0

0

0

0

0

0

0

0

0

0

0

0

0

0

0

0

0

0

0

0

0

0

0

0

8

8

8

8

0

0

0

0

0

0

0

1

1

1

0

0

0

1

1

0

0

0

0

0

0

0

0

0

0

0

0

0

0

0

3149

126

126

7

7

7

67

0

0

0

0

0

67

0

0

0

67

52

6

6

1

0

0

0

1

0

0

6

6

0

0

0

0

0

0

0

0

22

2

20

0

17

1

16

0

0

0

6

6

6

6

6

218

37

3

0

0

0

0

0

0

0

0

1

1

2

2

0

0

34

34

34

0

41

2

2

2

1

1

1

32

32

32

3

3

3

2

2

2

1

1

1

5

0

0

0

0

0

0

0

0

0

0

0

0

5

5

5

0

0

0

0

0

0

0

135

73

70

1

69

3

0

3

0

0

0

0

7

7

1

2

4

20

1

0

1

18

0

18

1

0

1

35

0

0

0

0

35

35

238

160

0

0

0

46

34

0

13

0

7

3

8

3

0

0

0

0

0

10

0

1

9

1

0

1

0

1

1

114

2

2

31

31

80

80

0

0

1

1

78

51

51

10

39

2

27

0

0

2

2

25

25

2555

0

0

0

0

0

0

0

0

0

0

0

0

0

0

0

0

0

0

0

0

4

4

0

0

0

0

0

0

0

0

0

1

1

0

0

1

0

0

0

1

2

0

2

0

0

0

0

0

0

0

0

0

0

0

0

0

0

0

0

0

0

0

66

19

1

0

0

1

0

18

18

3

3

0

0

0

3

44

34

4

30

0

0

10

10

0

0

111

111

0

0

0

111

0

71

1

1

2

17

0

19

0

0

8

2

0

0

2

2

0

0

3

0

0

3

3

0

0

0

0

1

1

1

1

1

0

1

1

0

0

0

0

1

1

1

0

0

0

0

0

0

1

0

0

1

1

0

147

147

0

0

0

0

147

23

0

0

0

0

8

0

0

0

0

15

101

0

1

1

1

1

0

0

0

1

1

0

0

1

0

0

0

0

1

0

0

534

519

5

5

0

0

0

0

0

0

42

0

42

57

57

2

0

2

405

18

387

0

8

8

15

2

0

1

1

0

0

10

0

0

0

0

0

0

2

2

0

0

0

0

0

0

5

1

0

0

0

0

0

0

0

3

3

0

0

0

0

0

10

10

0

0

0

0

1

0

0

0

1

0

0

9

1

1

1

0

5

1

0

0

0

0

0

0

0

0

0

0

0

1672

1259

0

0

1205

1205

10

0

10

1

1

0

0

0

32

0

21

2

6

3

0

0

2

2

9

9

8

0

0

2

2

0

0

4

1

3

2

0

2

0

0

0

0

0

0

0

2

2

2

0

0

0

0

401

0

0

2

2

0

0

3

1

2

0

0

0

0

0

0

1

1

0

0

0

0

0

0

0

0

395

0

41

354

0

0

0

0

0

0

0

0

0

0

0

2

0

0

2

2

6

6

6

6

0

2

3

1

951

951

951

951

0

0

951

23

5

19

14

110

157

0

0

0

70

247

0

65

0

40

6

46

40

109

0

0

0

0

0

0

0

0

0

0

0

0

0

0

0

0

0

0

227

227

227

227

227

227

1066

1066

1066

1066

47

47

0

0

0

0

1019

1019

0

0

0

0

0

0

0

0

0

0

0

0

62

62

62

62

20

5

11

4

42

42

0

0

0

0

0

0

0

0

0

0

0

0

0

0

1

0

0

0

0

0

0

0

0

0

0

0

0

0

0

0

1

1

0

0

0

0

0

0

1

1

1

3

3

3

3

3

2

1

0

0

45

25

5

5

5

5

20

2

2

1

1

0

0

0

0

0

0

18

2

2

4

4

0

0

12

12

12

10

10

10

2

6

2

0

0

0

0

0

0

0

0

2

2

0

0

1

0

0

1

0

0

0

1

0

0

1

0

0

0

0

1

0

0

0

0

1

1

1

1

0

0

0

0

1

1

1

0

0

1

0

1

0

0

0

0

0

0

0

0

0

0

0

6

6

1

1

1

1

1

1

4

4

4

0

0

0

0

156

0

0

0

0

0

0

0

0

0

0

0

0

0

0

0

0

0

0

0

0

0

0

0

0

0

0

0

0

0

0

0

0

0

0

0

0

0

0

0

0

0

0

0

0

0

0

0

0

0

0

0

0

0

0

0

0

0

0

0

0

0

0

0

0

0

0

0

0

156

156

13

13

13

3

3

3

14

14

14

0

0

0

0

0

0

0

0

125

44

44

2

0

2

8

8

0

0

71

71

0

0

0

0

0

1

1

1

0

0

0

0

0

0

0

0

0

0

0

0

0

0

0

2070

2070

2070

2070

0

0

2070

477

1387

206

0

0

0

0

6

4

0

0

0

0

0

0

0

0

0

0

0

4

2

2

2

0

0

0

0

0

0

1

1

1

1

1

1

0

0

0

2

2

2

2

2

0

0

0

0

0

0

0

0

0

0

0

0

0

0

0

0

0

0

0

0

0

0

0

0

0

0

0

0

0

0

0

0

0

2

2

2

2

1

1

1

1

30877

8235

21

21

0

0

0

1

1

20

0

19

0

0

0

1

3

3

0

0

3

3

257

1

1

1

0

0

0

256

16

16

240

240

14

14

14

2

12

0

2781

2522

1

1

2

2

0

0

0

0

0

115

30

85

0

0

0

77

75

0

2

0

0

3

2

1

9

9

0

0

0

0

1

1

40

40

3

0

3

0

29

0

0

29

0

0

54

0

7

1

0

5

1

0

0

0

26

1

0

7

0

0

0

5

0

1

0

1

1

0

0

46

1

45

0

0

49

0

44

5

480

139

7

198

131

0

5

44

0

0

0

2

41

0

1

0

0

1

1

91

19

0

0

68

4

135

135

36

36

1

1

186

186

0

0

0

0

0

0

0

0

27

27

10

10

138

0

12

99

27

0

0

1

1

84

84

4

4

5

5

7

6

1

0

0

0

99

28

71

2

2

0

741

7

539

3

50

2

113

9

5

0

8

0

5

0

0

0

2

2

2

257

257

0

257

4699

2449

1216

1216

455

4

113

338

0

0

0

9

9

10

10

0

0

0

758

747

0

0

0

11

1

1

0

0

0

2069

4

4

0

0

1928

1928

0

0

106

106

2

2

29

28

1

181

1

1

0

0

0

0

0

1

1

179

179

0

0

28

28

0

0

0

0

27

22

2

0

0

2

0

1

1

1

7

2

2

2

0

0

0

0

0

0

3

3

3

2

2

2

0

0

0

0

0

0

0

0

0

362

213

7

1

6

1

1

38

38

2

2

0

0

0

1

1

1

0

1

0

0

6

6

0

0

8

8

0

0

0

36

3

0

0

30

3

14

1

0

1

1

0

0

6

5

0

0

0

0

43

43

32

1

31

24

0

0

0

24

18

18

18

0

0

0

12

0

0

0

11

11

0

0

0

1

1

0

0

0

1

1

1

1

0

0

1

1

117

45

3

1

0

1

17

23

6

6

0

0

1

1

0

0

0

0

0

20

7

3

3

7

43

39

4

0

0

2

1

1

0

0

0

63

6

6

6

0

0

0

4

4

4

53

53

53

0

0

0

20908

0

0

0

0

125

37

0

0

0

0

0

0

6

5

0

0

1

0

5

1

4

16

16

9

9

1

1

0

0

88

0

0

7

7

0

0

0

81

2

1

0

1

0

2

0

0

1

36

9

3

5

0

0

15

6

8

8

8

8

21

21

0

0

0

1

1

20

20

431

248

0

0

0

0

181

181

50

37

0

0

0

13

0

0

0

0

0

8

6

2

9

9

183

15

15

24

24

28

5

23

7

0

7

9

8

1

3

3

0

0

55

55

5

0

5

16

16

9

9

2

2

4

4

6

6

0

0

0

0

0

0

0

0

494

171

1

1

0

0

170

1

169

323

323

323

0

0

1

1

1

0

1

7

7

0

0

0

0

7

1

0

0

0

1

0

0

0

0

2

0

0

0

0

0

0

0

0

0

1

0

1

0

0

1

0

0

0

0

0

0

0

0

0

7

6

6

6

0

0

0

1

1

1

0

0

0

1730

1730

1730

1729

1

18083

9

9

6

3

553

0

0

0

0

553

553

0

0

0

0

14852

14846

19

2069

658

0

9825

0

1266

19

849

1

139

0

1

6

6

0

6

6

0

6

2590

52

52

0

0

10

7

2

1

0

0

0

0

524

0

250

6

0

98

170

21

21

497

246

62

0

1

157

31

16

16

1463

1369

5

89

1

1

0

5

5

1

0

1

0

0

0

73

72

72

0

0

1

1

0

0

0

0

0

0

0

0

0

1

1

0

0

1

1

0

0

0

0

0

0

0

0

0

0

0

0

1

1

1

1

1

1733

0

0

0

0

0

0

1733

0

0

0

155

0

0

0

0

0

0

3

3

0

152

0

0

0

0

0

0

0

114

38

1574

1088

1088

0

0

0

486

2

1

482

1

0

0

0

4

4

4

0

0

0

0

0

39

0

0

0

0

0

0

0

0

0

0

0

0

0

0

0

0

0

0

0

0

0

0

0

0

0

0

0

0

0

0

0

0

0

0

0

0

0

0

0

0

38

5

5

5

5

23

23

0

0

0

0

0

0

23

23

0

0

0

0

0

0

0

10

10

10

10

0

0

0

0

0

0

0

0

0

0

0

0

0

0

1

1

1

1

1

7

7

7

7

7

7

0

0

0

0

0

0

0

0

0

0

0

0

0

0

6

6

6

0

0

0

1

1

1

5

5

5

0

0

0

0

0

0

0

0

0

0

0

0

0

0

0

0

0

0

0

0

0

0

0

0

0

0

0

0

0

0

0

0

0

0

0

0

0

0

0

0

0

0

0

0

0

0
